# The Occurrence of Putative Nitric Oxide Dismutase (Nod) in an Alpine Wetland with a New Dominant Subcluster and the Potential Ability for a Methane Sink

**DOI:** 10.1155/2018/6201541

**Published:** 2018-11-08

**Authors:** Yanfen Zhang, Anzhou Ma, Wenzong Liu, Zhihui Bai, Xuliang Zhuang, Guoqiang Zhuang

**Affiliations:** ^1^Key Laboratory of Environmental Biotechnology, Research Center for Eco-Environmental Sciences, Chinese Academy of Sciences, Beijing 100085, China; ^2^University of Chinese Academy of Sciences, Beijing 100049, China

## Abstract

Recently, a new oxygenic pathway has been proposed based on the disproportionation of NO with putative NO dismutase (Nod). In addition to a new process in nitrogen cycling, this process provides ecological advantages for the degradation of substrates in anaerobic conditions, which is of great significance for wastewater treatment. However, the Nod distribution in aquatic environments is rarely investigated. In this study, we obtained the *nod* genes with an abundance of 2.38 ± 0.96 × 10^5^ copies per gram of dry soil from the Zoige wetland and aligned the molecular characteristics in the corresponding Nod sequences. These Nod sequences were not only found existing in NC10 bacteria, but were also found forming some other clusters with Nod sequences from a WWTP reactor or contaminated aquifers. Moreover, a new subcluster in the aquifer-similar cluster was even dominant in the Zoige wetland and was named the Z-aquifer subcluster. Additionally, soils from the Zoige wetland showed a high potential rate (10.97 ± 1.42 nmol of CO_2_ per gram of dry soil per day) for nitrite-dependent anaerobic methane oxidation (N-DAMO) with low abundance of NC10 bacteria, which may suggest a potential activity of Nod in other clusters when considering the dominance of the Z-aquifer subcluster Nod. In conclusion, we verified the occurrence of Nod in an alpine wetland for the first time and found a new subcluster to be dominant in the Zoige wetland. Moreover, this new subcluster of Nod may even be active in the N-DAMO process in this alpine wetland, which needs further study to confirm.

## 1. Introduction

Photosynthesis is a widely known biological pathway that produces oxygen, but it is not the only pathway. In recent years, in addition to chlorate respiration [1] and detoxification of reactive oxygen species [2], a new oxygen-forming pathway has been proposed. The new pathway has the ability to produce oxygen in anaerobic conditions with nitrogen oxides (except N_2_O) as substrates [3], which is very favorable for the degradation and oxidation of organic matter in anaerobic environments rich in nitrogen oxides, such as wetlands and wastewaters.

The critical step in this pathway is the proposed disproportionation of NO with putative NO dismutase (Nod) [3–6]. Nod was first proposed in enrichment cultures dominated by “*Candidatus* Methylomirabilis oxyfera,” a representative species in the NC10 phylum [7, 8]. Currently, the NC10 bacteria have been reported to exist in various kinds of environments, such as lakes [9, 10], rivers [11], paddy fields [12], marine environments [13], and especially wetlands [14–16]. However, there are few reports of environmental Nod sequences. In a nitrite-dependent anaerobic methane oxidation (N-DAMO) reactor inoculated with river sediments, *nod* genes are first detected with specific primers [17]. Then, *nod* genes are reported to be abundant in contaminated aquifers and wastewater treatment systems [18]. In addition to these, *nod* transcripts in marine oxygen minimum zone water bodies were also reported [13].

In addition to NC10 bacteria, Nod is also speculated to exist in a facultatively denitrifying *γ*-proteobacterium strain HdN1, which can grow on alkanes from C6 to C30 (except hexadecane) with nitrate or nitrite in anaerobic conditions [5, 6, 19]. This means Nod exists not only in NC10 phylum but also in other microbes that can use oxygen for substrate activation [6]. The direct investigation of Nod will be more valuable than just the investigation of NC10, especially in understanding the environmental significance of this oxygenic pathway.

With the oxygen produced by Nod, NC10 bacteria have the ability to aerobically oxidize methane in anaerobic conditions with nitrite as the electron acceptor [3]. NC10 and “*Candidatus* Methanoperedens nitroreducens,” an archaeal group which oxides methane with nitrate as the electron acceptor, together perform the complete denitrifying anaerobic methane oxidation (DAMO). The DAMO process not only provides the unique link between the nitrogen and carbon cycles [20], but also has been considered as a solution to the sustainable operation of WWTP [21]. Therefore, the study of the key enzyme Nod in aquatic environments will not only promote the understanding of the metabolism of substances in the biogeochemical cycle, but also likely present better solutions for wastewater treatment operation.

Located on the Tibetan Plateau, the Zoige wetland is a typical alpine wetland and has large emissions of methane resulting from a huge carbon stock [22]. Although there is a prevalence of NC10 bacteria in wetlands [14–16], the presence and ecological significance of Nod in wetlands is still lacking. Here, we propose the following hypotheses: (1) Nod exists in this alpine wetland, and (2) microbes containing Nod may play a role in the methane sink of a natural wetland.

## 2. Materials and Methods

### 2.1. Sampling Methods

The samples used in this study were from the Zoige wetland, which is located on the Tibetan Plateau. Three sampling sites across the Zoige wetland were set in this study. For each sampling site, a five-point sampling method was used and sampling depths were 10 to 20 cm below the soil surface. For all the sampling sites, a depth of about 5 to 15 cm of standing water remained during the sampling period. The fresh soils were transported at 4°C to the laboratory. All experiments in this study were conducted in triplicates.

### 2.2. DNA Isolation

DNA was isolated from soils using a method described before [23] with slight modifications. The Lysing Matrix E tubes from MP Biomedicals were used with FastPrep-24 from the same company for the lysing step. Before the total nucleic acids were precipitated, RNase stock solution was added with a final concentration of 10 *μ*g/ml. After being incubated at 37°C for at least one hour, the added enzyme was removed by repeated mixing of chloroform-isoamyl alcohol (24 : 1) and repeated centrifugation. The quality of the obtained DNA solutions was checked by agarose gel analysis, and the concentration was measured with a Nanodrop® ND-1000 UV-Vis spectrophotometer (Nanodrop Technologies, Wilmington, DE, USA).

### 2.3. PCR and qPCR

DNA samples diluted 5- or 10-fold were used as templates for PCR and qPCR analyses. Primer pairs used for PCR and qPCR of the *nod* gene were nod684Fv2/nod1706Rv2 and nod1446F/nod1706Rv2, respectively [18]. The primer pair used for qPCR of NC10 16S rRNA gene was qP2F/qP2R [8]. The PCR reactions were performed using a volume of 26 *μ*l with the following composition: 22 *μ*l of Golden Star T6 Super PCR Mix (1.1x) (Beijing TsingKe Biotech Co. Ltd.), 1 *μ*l of each primer (10 *μ*M), and 2 *μ*l of template DNA. The program was as follows: 98°C for 2 min, followed by 37 cycles of 98°C for 15 s, 57°C for 20 s, and 72°C for 30 s. Then, there was a final 5 min extension at 72°C. The sequences from qPCR products were verified by cloning and sequencing in pLB vector and were then used as standards for *nod* gene and NC10 16S rRNA gene. The standard curve concentrations for *nod* gene were from 2.580 × 10^3^ to 2.580 × 10^10^ copies per gram of soil. The standard curve concentrations for NC10 16S rRNA gene were from 2.183 × 10^3^ to 2.183 × 10^10^ copies per gram of soil. A Bio-Rad CFX Connect™ Real-Time PCR Detection System and SYBR® Premix Ex Taq™ (Tli RNaseH Plus) were employed in the qPCR reactions. The qPCR reactions were performed using a volume of 25 *μ*l with the following composition: 12.5 *μ*l of SYBR Premix Ex Taq (Tli RNaseH Plus) (2x), 0.5 *μ*l of each primer (10 *μ*M), 2 *μ*l of the DNA template, and 9.5 *μ*l of sterile distilled water. The qPCR program was as follows: 95°C for 30 s, followed by 40 cycles of 95°C for 5 s, and 60°C for 30 s. Then, a melt curve was performed with 95°C for 5 s and 60°C to 95°C increasing at a rate of 0.5°C/5 s. The standards and samples were quantified in triplicate, and the analysis was performed with an efficiency of 100 ± 10%.

### 2.4. Cloning, Sequencing, and Phylogenetic Analysis

PCR products of the *nod* gene were purified with a TIANgel Midi Purification Kit (TIANGEN, Beijing) according to the manufacturer's protocol. Purified PCR products were cloned using a Lethal Based Fast Cloning Kit (TIANGEN, Beijing). The colonies were detected by PCR and agarose gel analysis for positive colonies, and then the PCR products of positive colonies were sent to sequencing. The high-quality sequences obtained were assigned to the same operational taxonomic units (OTUs) by Mothur based on a cutoff of 0.03. Also, the rarefaction curve was also calculated with Mothur. Then, the representative sequences of each OTU were translated to amino acids and aligned with selected qNor, cNor, and some published Nod sequences using a previously published method [18]. Then, based on the alignment file, a phylogenetic tree was constructed with MEGA7 using the neighbor-joining method.

### 2.5. Incubation Experiments

In an anaerobic box, soils were mixed with sterile anaerobic distilled water in a volume ratio of 1 : 4, and the roots in the slurry were removed. Then, the slurry was split into 120 ml glass vials with 10 ml of slurry in each bottle. After being sealed with butyl rubber stoppers and aluminum caps, these bottles were taken out of the anaerobic box. Then, the bottles were vacuumed and flushed with high-purity argon for 5 min in five cycles. After the final step of the flush, the pressure in the headspace gas was balanced by a syringe. Then, these bottles were preincubated at 14°C for 116 days. After preincubation, NaNO_2_ was added with a final concentration of 200 *μ*M in triplicate bottles, and sterile anaerobic distilled water was used for the control. After the headspaces of these bottles were all vacuumed and flushed like before, 5 ml of gas of the headspace was replaced by an equal volume of ^13^CH_4_ (99.9% purity, 99.8% atom% ^13^C). Samples were incubated at 14°C. The production of ^13^CO_2_ was measured by a gas chromatograph mass spectrometer (GCMS-QP2010 Ultra, Shimadzu). The rates were calculated by a linear regression of the produced ^13^CO_2_ over time.

### 2.6. Statistical Analyses

All statistical analyses in this article were conducted using SPSS software (PASW Statistics 18, IBM, USA). The significance of the difference between the abundance of NC10 16S rRNA gene and *nod* gene was performed by a nonparametric test. The significance of difference between rates in different treatments was calculated by a general linear model (univariate).

## 3. Results and Discussion

**Figure 1:**
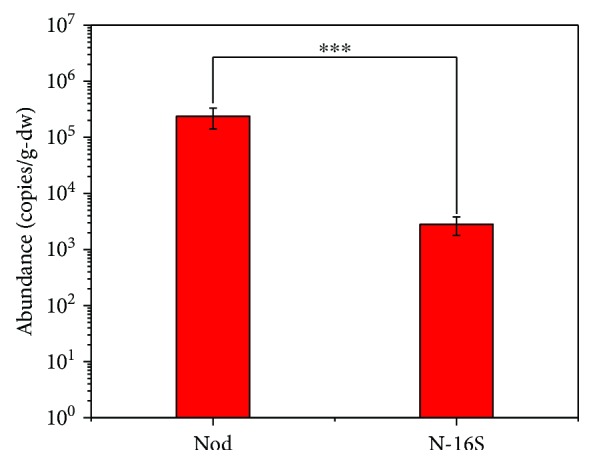
Abundance of NC10 16S rRNA and *nod* genes in soil from the Zoige wetland.

**Figure 2:**
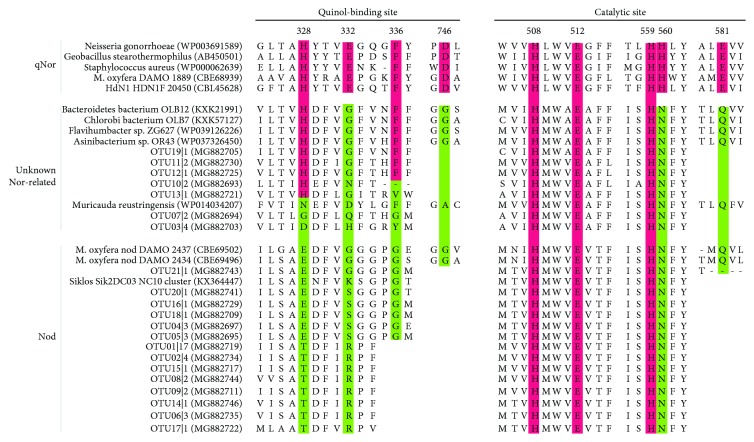
Alignment of the quinol-binding site and the catalytic site in Nod sequences and qNor sequences. The conserved residuals in qNor are highlighted in red, and the substitutions in putative Nod and putative Nor are shown in green.

The physiochemical properties of soils from the Zoige wetland are shown in Table 1. The results of the quantification showed that we obtained the amplification products of *nod* genes in the Zoige wetland with an abundance of 2.38 ± 0.96 × 10^5^ copies per gram of dry soil (Figure 1). Since Nod is proposed based on quinol-dependent NO reductase (qNor) paralogs, it has a close phylogenetic distance with canonical qNor, and the characteristics for Nod are several amino acid substitutions in the quinol-binding sites and catalytic sites, which are essential for the canonical qNor [6, 24]. The Nod sequences (translated from *nod* sequences) recovered in the Zoige wetland also had substitutions in these key amino acid sites, which are similar to those in *M. oxyfera* (Figure 2). These results suggest the actual occurrence of Nod in the Zoige wetland.

**Figure 3:**
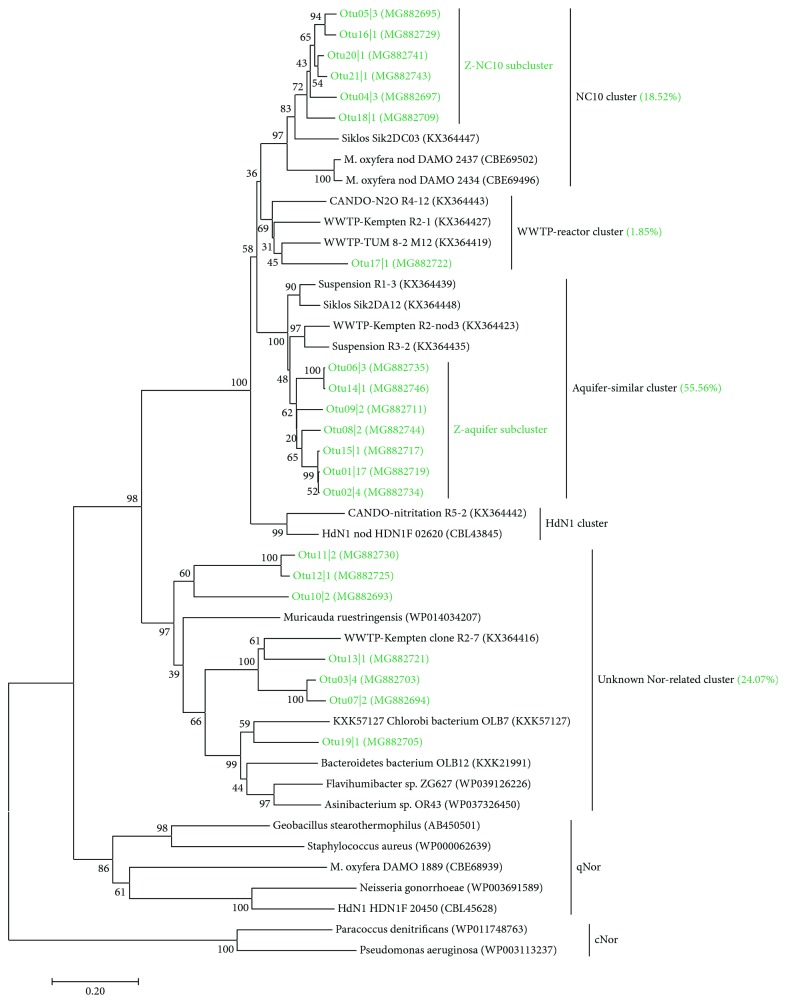
Phylogenetic tree of putative Nod obtained in this study with selected qNor, cNor, and Nod sequences. The accession numbers of the reference sequences and representative *nod* sequences obtained in this study are shown in parentheses. The Nod sequences obtained in this study are shown in green. The relative abundance of the four clusters obtained in this study are shown in parentheses after the names of the clusters.

The results of the quantification also verified the existence of NC10 bacteria with an abundance of 2.80 ± 1.02 × 10^3^ copies per gram of dry soil, which is significantly lower than that of *nod* genes (Figure 1). This may be the result of more *nod* gene copy numbers than 16S rRNA in one single cell or the *nod* genes may exist in microbes other than NC10 bacteria. After phylogenetic analysis of the obtained 54 high-quantity Nod sequences (21 OTUs), we found that there are partial sequences that have large distances with the published *M. oxyfera* Nod sequences (Figure 3). All the sequences obtained from the Zoige wetland formed three clusters with published Nod sequences and one cluster with some unknown Nor-related sequences, which were all distinct from qNor and cNor (Figure 3). The three clusters of Nod were named after the closely related published Nod sequences [18], namely, the NC10 cluster, the aquifer-similar cluster, and the WWTP-reactor cluster. In addition, Nod sequences from the Zoige wetland in the NC10 cluster and the aquifer-similar cluster even formed subclusters, which were named as Z-NC10 subcluster and Z-aquifer subcluster, respectively. The sequences in the aquifer-similar cluster and the WWTP-reactor cluster were not only distinct from the NC10 Nod clusters but also had distances with the HdN1 Nod cluster. Moreover, in the alignment with qNor sequences, substitutions of the His328 and Glu332 sites in the Nod sequences of the aquifer-similar cluster and the WWTP-reactor cluster were different from those in the NC10 Nod sequences (Figure 2). These results suggest that the Nod sequences in the aquifer-similar cluster and the WWTP-reactor cluster are from microbes other than NC10 and HdN1. In addition, these unknown microbes were more abundant than NC10 in the Zoige wetland according to the relative abundance of each cluster (Figure 3).

**Figure 4:**
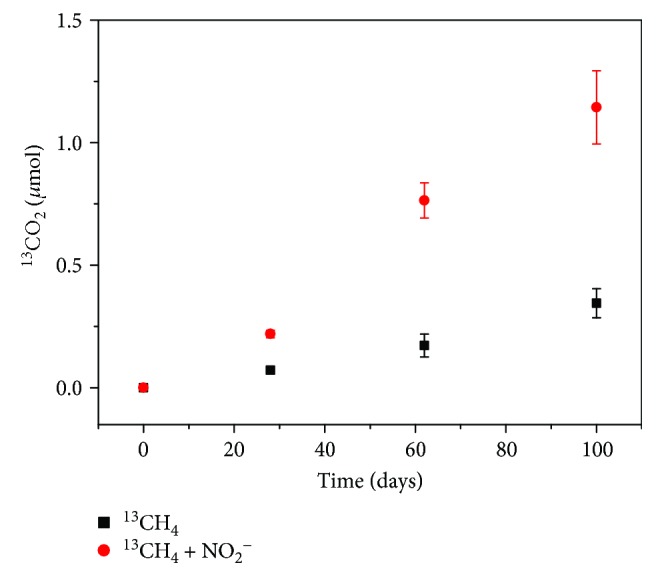
The production rate of ^13^CO_2_ from ^13^CH_4_ in the incubation of soils from the Zoige wetland.

With the activity of Nod, NC10 has the ability to oxide methane in anaerobic conditions with nitrite as the electron acceptor. To test the N-DAMO activity of soils from the Zoige wetland, ^13^CH_4_ was added to trace the methane oxidation process. The result showed that the methane oxidation rate with nitrite as the electron acceptor was 15.39 ± 1.29 nmol of CO_2_ per gram of dry soil per day (*R* ^2^ = 0.97), which is significantly higher (*P* ≤ 0.01) than the methane oxidation rate in the control (4.43 ± 0.43 nmol of CO_2_ per gram of dry soil per day (*R* ^2^ = 0.97)) (Figure 4). This suggests a significant N-DAMO activity in soils from the Zoige wetland. After calculation, the net oxidation rate of methane was 10.97 ± 1.42 nmol of CO_2_ per gram of dry soil per day (*R* ^2^ = 0.95). According to a previous report [25], the mean methane flux in the Zoige wetland was approximately 2.43 mg m^−2^ h^−1^. The density of soils from the Zoige wetland was measured to be 0.31 g/ml in the current study. Assuming that the active layer of N-DAMO was only in the depth of 10–20 cm, which is the sampling depth in the current study, the net N-DAMO was about 0.23 mg m^−2^ h^−1^, which is about 9.5% of the reported methane fluxes. In addition, the N-DAMO rate in our study is similar to that of a minerotrophic peatland [14] and even higher than the rate in an urban wetland [26] and some other wetlands [15]. However, the abundance of NC10 in the Zoige wetland (2.80 ± 1.02 × 10^3^) is much lower than its abundance in all these wetlands, which is approximately 10^6^–10^7^ copies per gram of soil. Moreover, in these previous reports [15, 26], N-DAMO activity was usually not detected when the abundance of NC10 declined close to 10^5^ copies per gram of soil. Therefore, the high N-DAMO rate in the current study may not only be performed by the NC10 bacteria, especially considering the dominance of new subclusters in the Zoige wetland (Figure 3). This means that the Nod in some unknown microbes may also be active in utilizing nitrous oxides and may even play a role in coupling carbon and nitrogen cycling. This speculation needs further studies to confirm, such as the analysis of the ^13^C-labeled DNA in the N-DAMO process.

## 4. Conclusion

This study revealed the occurrence of *nod* gene in an alpine wetland for the first time with an abundance of 2.38 ± 0.96 × 10^5^ copies per gram of dry soil. In addition to the reported Nod in NC10 bacteria, there were some different Nod sequences and one of a subcluster (Z-aquifer subcluster) that was even dominant in the Zoige wetland. Moreover, soils from the Zoige wetland exhibited a high N-DAMO rate with a low abundance of NC10 bacteria, which may mean a potential ability of Nod in other clusters to oxide methane in an anaerobic condition. However, this speculation needs further work to confirm.

## Figures and Tables

**Table 1:** Physiochemical properties of soils from the Zoige wetland.

Samples	T (°C)	pH	Water content (%)	SO_4_^2−^ (mg/l)	NH_4_^+^ (mg/l)	NO_3_^−^ (*μ*g/l)
Site1	13.0	7.42 ± 0.03	0.71 ± 0.02	5.69 ± 0.43	5.52 ± 0.94	13.33 ± 3.56
Site2	13.5	7.28 ± 0.02	0.73 ± 0.01	3.17 ± 0.68	4.08 ± 0.14	17.56 ± 0.58
Site3	13.5	7.31 ± 0.02	0.71 ± 0.01	2.26 ± 0.26	4.51 ± 0.97	27.67 ± 4.59

## Data Availability

The representative nod sequences and nor-related sequences obtained in this study were deposited at NCBI under the accession numbers MG882693~MG882746. It is also available from the corresponding author upon request.

## Supplementary Materials

Supplementary Materials Figure S1: rarefaction curve for the nod gene library in this study.
