# Supplementary material for: The Occurrence of Putative Nitric Oxide Dismutase (Nod) in an Alpine Wetland with a New Dominant Subcluster and the Potential Ability for a Methane Sink
Source: Archaea. 2018 Nov 8;2018:6201541. doi: 10.1155/2018/6201541 (PMC6250046; doi:10.1155/2018/6201541)

The occurrence of putative nitric oxide dismutase (Nod) in an alpine wetland with a new dominant sub-cluster and the potential ability for a methane sink

Yanfen Zhang[a,b], Anzhou Ma[a,b], Wenzong Liu[a,b], Zhihui Bai [a,b], Xuliang Zhuang[a,b], Guoqiang Zhuang[a,b*]

[a] Key Laboratory of Environmental Biotechnology, Research Center for Eco-Environmental Sciences, Chinese Academy of Sciences, Beijing 100085, China

[b] University of Chinese Academy of Sciences, Beijing 100049, China

Correspondence should be addressed to Guoqiang Zhuang, gqzhuang@rcees.ac.cn

Figure S1: Rarefaction curve for the *nod* gene library in this study.

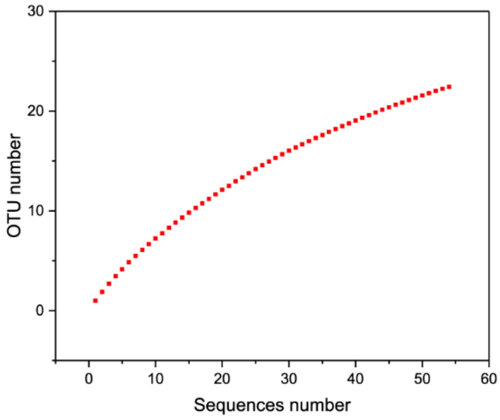

Supplement: Supplementary Materials — Figure S1: rarefaction curve for the nod gene library in this study. [file 6201541.f1.pdf]
